# Regulation of photosynthesis and stomatal and mesophyll conductance under water stress and recovery in olive trees: correlation with gene expression of carbonic anhydrase and aquaporins

**DOI:** 10.1093/jxb/eru160

**Published:** 2014-05-05

**Authors:** Alfonso Perez-Martin, Chiara Michelazzo, Jose M. Torres-Ruiz, Jaume Flexas, José E. Fernández, Luca Sebastiani, Antonio Diaz-Espejo

**Affiliations:** ^1^Group of Irrigation and Crop Ecophysiology, Instituto de Recursos Naturales y Agrobiología, IRNAS-CSIC, Apartado 1052, 41080, Sevilla, Spain; ^2^Biolabs, ISV, Scuola Superiore Sant’Anna, Piazza M. della Libertà 33, 56127 Pisa, Italy; ^3^Research Group in Plant Biology under Mediterranean Conditions, Universitat de les Illes Balears; Carretera de Valldemossa Km 7.5, 07122 Palma de Mallorca, Illes Balears, Spain

**Keywords:** AQP, CA, mesophyll conductance, stomatal conductance, *OePIP*, *Olea*, photosynthesis limitations, water stress.

## Abstract

The hypothesis that aquaporins and carbonic anhydrase (CA) are involved in the regulation of stomatal (*g*_s_) and mesophyll (*g*_m_) conductance to CO_2_ was tested in a short-term water-stress and recovery experiment in 5-year-old olive plants (*Olea europaea*) growing outdoors. The evolution of leaf gas exchange, chlorophyll fluorescence, and plant water status, and a quantitative analysis of photosynthesis limitations, were followed during water stress and recovery. These variables were correlated with gene expression of the aquaporins *OePIP1.1* and *OePIP2.1*, and stromal CA. At mild stress and at the beginning of the recovery period, stomatal limitations prevailed, while the decline in *g*_m_ accounted for up to 60% of photosynthesis limitations under severe water stress. However, *g*_m_ was restored to control values shortly after rewatering, facilitating the recovery of the photosynthetic rate. CA was downregulated during water stress and upregulated after recovery. The use of structural equation modelling allowed us to conclude that both *OePIP1.1* and *OePIP2.1* expression could explain most of the variations observed for *g*_s_ and *g*_m_. CA expression also had a small but significant effect on *g*_m_ in olive under water-stress conditions.

## Summary

In plants with sclerophyll leaves, the response of stomatal and mesophyll conductance to CO_2_ to water stress and recovery is correlated with the expression of aquaporins and carbonic anhydrase.

## Introduction

Water stress is considered the main environmental factor limiting photosynthesis, plant growth, and yield worldwide, especially in semi-arid areas, where *Olea europaea* is well adapted (Boyer, 1982; Lawlor, 1995; Flexas *et al.*, 2004). Under water-stress conditions, which are related to water depletion and/or high atmospheric vapour pressure deficit (VPD), photosynthesis decreases through several mechanisms including stomata closure, reduced mesophyll conductance to CO_2_ (*g*_m_), and feedback regulation by end-product accumulation (Nikinmaa *et al.*, 2013). As soil water deficit and VPD result in reduced stomatal conductance to CO_2_ (*g*_sc_) (reviewed by Lawlor & Cornic, 2002; Flexas *et al.*, 2004) and *g*_m_ (Flexas *et al*., 2002, 2004; Centritto *et al.*, 2003; Galmés *et al.*, 2007a; Warren, 2008*a*; Peeva and Cornic, 2009) in many species, several authors have suggested a possible co-regulation of *g*_sc_ and *g*_m_ (Centritto *et al.*, 2003; Flexas *et al*., 2002, 2012). However, such co-regulation depends on the species and the prevailing conditions, such as the combination of drought with VPD (Perez-Martin *et al.*, 2009) or with radiation (Flexas *et al.*, 2009; Galle *et al.*, 2009).

Under natural conditions, water stress normally develops gradually over periods of weeks or months, and hence it is possible that some acclimation occurs in addition to day-to-day variations in response to variable environmental conditions (Flexas *et al.*, 2006*a*). Acclimation to water stress may comprise responses involving modification of gene expression and plant physiology and morphology, taking place over days to weeks, which lead to a homeostatic compensation for the initial negative effects of water stress on photosynthesis (Lambers *et al.*, 2008). Nonetheless, little is known about the acclimation of photosynthesis to water-stress conditions in the short term, so studies that specifically address this issue are needed (Flexas *et al.*, 2006*a*). The recovery phase after relief of stress (i.e. rainfall or irrigation) becomes another important part of the overall plant physiological response to a water-stress period. The capability for photosynthetic recovery from a water-stress period determines the future growth and survival of plants in their habitat, and depends on the degree and velocity of photosynthesis decline during water depletion (Flexas *et al*., 2006*a*, 2009). Although there is much information on the regulatory mechanisms of the response of *g*_s_ to water stress and recovery (Buckley, 2005), little is known about the regulation of *g*_m_ (Flexas *et al.*, 2008). The main limiting factor for photosynthesis during water stress or recovery can vary depending on species (Galmés *et al.*, 2007*a*; Ennahli and Earl, 2005), the intensity of previous stress (Flexas *et al.*, 2009), light and temperature (Galle *et al.*, 2009), plant age (Varone *et al.*, 2012), and the application of successive drought and recovery cycles (Galle *et al.*, 2011).

Therefore, besides photosynthetic biochemistry, the regulation of *g*_s_ and *g*_m_ is crucial to understand both processes of acclimation to stress and recovery after stress. Despite being one of the most studied physiological variables, the regulation of stomata is not yet fully understood (Buckley and Mott, 2013). There is, however, a large consensus on the fact that two main components operate to produce the adequate response of *g*_s_ to environmental stimuli: a hydropassive loop, related to the hydraulic capacity of the plant, and a hydroactive loop, linked to both the chemical signalling and the photosynthetic capacity of the leaf (Buckley *et al.*, 2003). Regarding the regulation of *g*_m_, recent evidence suggests that anatomical traits, such as cell-wall thickness and chloroplast distribution, are among its stronger determinants (Flexas *et al.*, 2012; Tosens *et al.*, 2012; Tomás *et al.*, 2013). Nevertheless, the most likely candidates proposed for the rapid regulation of *g*_m_ are aquaporins (AQPs) and carbonic anhydrase (CA). There are several pieces of evidence of AQP involvement in *g*_m_ regulation. First, inhibiting the activity of some aquaporins by HgCl_2_, Terashima and Ono (2002) found a decreased *g*_m_ in *Vicia faba*. Secondly, the tobacco aquaporin NtAQP1 facilitates CO_2_ membrane transport when inserted in *Xenopus* oocytes (Uehlein *et al.*, 2003). Finally, the strongest evidence comes from the observed altered *g*_m_ in transformed and mutants plants (Hanba *et al.*, 2004; Flexas *et al.*, 2006*b*; Heckwolf *et al.*, 2011; Kawase *et al.*, 2013).

On the other hand, as aquaporins accumulate in cells around stomatal cavities and in guard cells themselves (Otto & Kaldenhoff, 2000), they may also be involved in the regulation of *g*_s_. In fact, transgenic and mutant plants with altered AQPs present differences not only in *g*_m_ but also in *g*_s_ (Hanba *et al.*, 2004; Flexas *et al.*, 2006*b*; Heckwolf *et al.*, 2011). Recently, Pou *et al.* (2013) found a strong correlation between *g*_s_ and the gene expression of some particular AQPs during water stress and recovery in grapevines. Despite all these findings, the potential relationship between the gene expression of AQPs and the variations in *g*_s_ and *g*_m_ is still unclear, as patterns of AQP expression are complex and different AQP forms may induce distinct responses (Tyerman *et al.*, 2002; Alexandersson *et al.*, 2005, Galmés *et al.*, 2007*b*)

Concerning the involvement of CA in the regulation of *g*_m,_ less evidence has been found. CA might have a role in the regulation of *g*_m_ through the establishment of the dynamic equilibrium between CO_2_ and $HCO_{3}^{–}$ (Tiwari *et al.*, 2005; Tholen and Zhu, 2011; Flexas *et al.*, 2012). Likewise, the presence of isoforms of CA in all the compartments composing the diffusion way of the liquid phase of CO_2_ (except on the cellular wall) suggests that they may be involved somehow in the regulation of *g*_m_ (Evans *et al.*, 2009; Terashima *et al.*, 2011). The elimination of the stroma CA might result in a 50% reduction in *g*_m_ (Tholen and Zhu, 2011) but, in contrast, studies with mutant plants with reduced CA have shown little reduction in *g*_m_ (Price *et al.*, 1994; Williams *et al.*, 1996). Some authors have claimed that the role of CA could be species dependent and become more important in species with a high CO_2_ resistance in their cell walls and low *g*_m_, as is the case of many drought-adapted species (Gillon and Yakir, 2000).

*Olea europaea* is an excellent plant model for studying physiological and molecular responses to water stress and recovery under realistic water-stress conditions because of its high reputation as drought-tolerant species (Fernández and Moreno, 1999; Connor, 2005), as well as its importance in the Mediterranean landscape and agronomical impact (Sofo *et al.*, 2009; Boughalleb and Hajlaoui, 2010). As no previous study has related AQP and CA gene expression with all the potential photosynthesis-limiting factors (*g*_s_, *g*_m_ and biochemistry) during water stress and recovery under field conditions, we studied the expression of two AQP genes characterized in olive, *OePIP1.1* and *OePIP2.1*, and a stromal CA. The experiment was carried out outdoors, under natural conditions of radiation and atmospheric demand, in contrast to most up-to-date studies of the relationships between AQPs and CA, which have been performed in plants grown in growth cabinets. The specific objectives of the present work were: (i) to quantify the photosynthesis limitations imposed by *g*_s_, *g*_m_ and photosynthetic capacity during the water-stress and recovery period, and (ii) to relate these changes to changes in the gene expression of AQPs and CA in order to expand current knowledge on the possible role of the AQP and CA expression in the regulation of *g*_s_ and *g*_m_.

## Materials and methods

### Plant material and water-stress treatments

Eighteen 5-year-old *O. europaea* L. var. Manzanilla plants were grown outdoors in 50 l pots (see Supplementary Fig. S1 at *JXB* online) at ‘La Hampa’, an experimental farm of the Consejo Superior de Investigaciones Científicas (37°17′ N, 6°3′ W, altitude 30 m), near Seville (south-western Spain). Pots contained sandy-loam soil, typical of this farm and whose hydraulic properties have been described previously by Palomo *et al.* (2002). The experiment was performed during the summer of 2009. The climate was typically Mediterranean, with a mild, wet season from October to April and hot and dry conditions from May to September (Fernández *et al.*, 2006). Six trees were pruned by one-third of their leaf area to reduce leaf water demand and to produce a slower evolution of water stress. Branches were cut at dawn after being sprayed with water and were covered with plastic bags to minimize embolism risk. Pruning cuts were covered with wound dressing. Thus, two different water-stress intensities were imposed by withholding irrigation in six non-pruned trees (S) and six pruned trees (SP) on 26 July. Another group of six olive trees was maintained as well watered and was used as control treatment (C). Finally, after 13 d of total water withholding, S and SP plants were rewatered daily for the following 6 weeks and allowed to recover. All the physiological measurements detailed below were taken in leaves of a similar age, including those carried out during the recovery experiment. This ruled out the possibility that new, recently grown leaves were measured.

### Climatic variables and soil water status

Meteorological variables were measured with an automatic weather station (Campbell Scientific Ltd, Shepshed, UK) located 50 m away from the experimental trees. Average values taken over 30min of net radiation, global radiation, photosynthetically active radiation, wind speed, air temperature, relative humidity, and VPD were recorded.

The volumetric soil water content (*θ*, mm) of the substrate in the pots was determined by the time–domain–reflectrometry technique using a Tektronix cable tester (Model 1502C; Beaverton, OR, USA). Measurements were made at 7.00 Greenwich Mean Time (GMT) and averaged between 0.05 and 0.20 m in depth because there were no differences between the values recorded at either depth within each treatment. *θ* was transformed into soil relative extractable water (REW) according to Fernández *et al*. (1997):

$$\;\text{REW}=(\theta -\theta_{\text{min}})/(\theta_{\text{max}}-\theta_{\text{min}}),$$

where *θ* is the actual soil water content, *θ*_min_ is the minimum soil water content measured during the experiment (mm), and *θ*_max_ is the soil water content at field capacity (mm). Field capacity was determined to be 0.25 m^3^ m^–3^ and the soil water content at permanent wilting point was 0.04 m^3^ m^–3^. A REW value of >1 could be observed for several hours after water application, especially in the C treatment, as during that period *θ* exceeded field capacity.

### Plant water relationships

Leaf water potential was assumed to be equal to xylem pressure potential at the petiole in transpiring leaves and was measured in six leaves per treatment at pre-dawn (*Ψ*_pd_) and 11.00 GMT (*Ψ*) with a pressure chamber (Soilmoisture Equipment, Santa Barbara, CA, USA). The relative water content of leaves (RWC) was determined at 11.00 GMT in opposite leaves to those sampled to measure *Ψ* as:

$$\begin{array}{l} \text{RWC}=\left(\text{fresh weight }–\text{ dry weight}\right)\\ \;/\left(\text{turgid weight }–\text{ dry weight}\right)\times \text{1}00. \end{array}$$

Olive leaves were weighed immediately on harvest to determine fresh weight. To determine the turgid weight of the samples, these were kept in distilled water in darkness at 4 °C to minimize respiration losses until they reached a constant weight (full turgor, typically after 24h). Their dry weight was obtained after 48h at 70 °C in an oven.

### Leaf gas exchange and chlorophyll fluorescence

Leaf gas exchange in leaves similar to those used for plant water relationships was determined simultaneously with chlorophyll fluorescence at 11.00 GMT using the open gas-exchange system Li-6400 (Li-Cor, Lincoln, NE, USA) equipped with an integrated fluorescence chamber head (Li-6400–40; Li-Cor). Photosynthesis was induced with saturating light (1800 µmol m^–2^ s^–1^) and an ambient concentration of CO_2_ of 400 µmol mol^–1^.

From the fluorescence measurements, the actual photochemical efficiency of photosystem II (Φ_PSII_) was determined according to Genty *et al.* (1989) as:

$$\Phi_{\text{PSII}}=\left(F_{\text{m}}{}^{\prime }-F_{\text{s}}{}^{\prime }\right)/F_{\text{m}}{}^{\prime },$$

where *F*_s_ is the steady-state fluorescence in the light [here photosynthetic photon flux density (PPFD) of 1800 µmol m^–2^ s^–1^] and *F*_m_′ is the maximum fluorescence obtained with a light-saturating pulse (~8000 µmol m^–2^ s^–1^). As Φ_PSII_ represents the number of electrons transferred per photon absorbed by PSII, the rate of electron transport (*J*) can be calculated as:

$$J=\Phi_{\text{PSII}}\times \text{PPFD}\times 0.\text{5}\times 0.\text{93},$$

where PPFD is the PPFD incident on the leaf, 0.5 is a factor that assumes equal distribution of energy between the two photosystems (Laisk and Loreto, 1996), and 0.93 is the leaf absorptance determined in five leaves per treatment using an integrating sphere with a portable spectroradiometer (LI-1800; Li-Cor) and calculating absorptance as 1 – reflectance – transmittance. The relationship between Φ_PSII_ and the quantum efficiency of gross CO_2_ fixation (Φ_CO2_) was obtained by varying the light intensity under non-photorespiratory conditions in an atmosphere containing <1% O_2_ (Valentini *et al.*, 1995). The correlation between Φ_PSII_ and Φ_CO2_ was done under different conditions of soil water deficit and VPD (see Supplementary Fig. S2 at *JXB* online) to check for potential errors in the estimation of *g*_m_ due to non-linearity of electron transport rate.

Estimation of *g*_m_ was performed with the ‘variable *J* method’ of Harley *et al.* (1992):

$$g_{\text{m}}=A_{\text{N}}/\left\{C_{\text{i}}-(\Gamma *\left[J+\text{8}\left(A_{\text{N}}+R_{\text{d}}\right)\right]/\left[J\text{ 4}\left(A_{\text{N}}+R_{\text{d}}\right)\right]\right\},$$

where *A*_N_ and internal CO_2_ concentration (*C*_i_) were taken from gas-exchange measurements at saturating light, *J* was estimated from fluorescence, the rate of mitochondrial respiration in the light (*R*_d_) was assumed to be the same as measured dark respiration (Warren, 2004),and Γ* was estimated at the measuring temperature by using the kinetic constants proposed for this parameter by Bernacchi *et al.* (2002). To estimate *R*_d_, we covered the leaves (*n*=6) for 30min and measured the net assimilation of CO_2_ rates using a modified 2×3cm broadleaf chamber and an integrated light source (LI-6400-02B; Li-Cor) at a flow rate of 250 µmol air s^–1^. The relationships between Φ_PSII_ and Φ_CO2_, and *R*_d_ were measured in three stages of the experiment: one at the beginning of the experiment, the second 7 d after withholding irrigation, and the third 2 d after resuming irrigation. No significant differences in leaf absorptance were found throughout the experiment, despite changes in the RWC of leaves.

### Quantitative photosynthesis limitations analysis

To assess the limitations imposed by water stress and recovery on photosynthesis, a quantitative limitation analysis of photosynthesis was conducted according to Grassi and Magnani (2005), with modifications. This approach requires measurements of *A*_N_, *g*_s_, *g*_m_, and *V*_cmax_ to calculate the partition of photosynthesis limitations into components related to stomatal conductance (*S*_L_), mesophyll conductance (*MC*_L_), and leaf biochemical characteristics (*B*_L_), assuming a reference treatment where maximum assimilation rate, *g*_s_, *g*_m_, and *V*_cmax_ can be defined (see Supplementary Information in *JXB* Online for details of equations used). Total limitations (*T*_L_) were defined as the sum of *S*_L_, *MC*_L_, and *B*_L_. As actual electron transport rate (ETR, i.e. fluorescence-derived *J*) is tightly coupled with *V*_cmax_ (Galmés *et al.*, 2007*a*) and should indeed reflect gross photosynthesis (Genty *et al.*, 1989; Valentini *et al.*, 1995), *B*_L_ was calculated using ETR instead of *V*_cmax_ as a surrogate for leaf biochemistry. There were three reasons for the use of ETR as a surrogate of *V*_cmax_ in the limitation analysis: (i) under our experimental approach, it was unfeasible to build a number of *A*_N_–*C*_i_ curves enough to estimate *V*_cmax_ for every sampling date; in addition to this, when *g*_s_ is low, the estimation of *V*_cmax_ is highly prone to errors due to the low rates of leaf gas exchange; (ii) as actual electron transport rate is tightly coupled with *V*_cmax_ (Galmés *et al.*, 2007*a*) and should indeed reflect gross photosynthesis (Genty *et al.*, 1989; Valentini *et al.*, 1995), ETR can be used instead of *V*_cmax_ as a surrogate for leaf biochemistry; the uncertainties in the determination of *V*_cmax_ reported by several authors (Patrick *et al.*, 2009; Gu *et al.*, 2010) can be avoided by using ETR, as has been verified and confirmed by Galle *et al.* (2009, 2011); and (iii) we checked that, under our conditions and during the progress of water stress and recovery, there was a good correlation between ETR and *V*_cmax_ for the range measured; this correlation is shown in Supplementary Fig. S3 at *JXB* online. In the current study, the maximum assimilation rate, concomitantly with *g*_s_, *g*_m_, and ETR, was reached under well-watered conditions, and therefore the C plants were used as a reference. However, as *A*_N_ of the C plants increased during the experiment, presumably due to environmental changing conditions, the values of the C plants for each day were considered as the reference for the S and SP treatments determined on the same day.

### RNA extraction and real-time reverse transcription-PCR analysis

The same leaves used throughout the experiment for gas exchange and chlorophyll fluorescence measurements were harvested at 12.00 GMT for gene expression analysis. They were harvested at the same time of the day to avoid diurnal fluctuations in the transcripts (Henzler *et al.*, 1999; Laur and Hacke, 2013). Three replicates per treatment and day were sampled and were frozen immediately in liquid nitrogen. Leaf tissues of each sample were ground to a fine powder with liquid nitrogen using sterile mortars and pestles. RNA extraction was performed on 100mg of plant material using an RNeasy Plant Mini kit (Quiagen, Venlo, The Netherlands) and treated with RNase-Free DNase (Qiagen) to avoid genomic DNA contamination during RNA purification. The concentration of total RNA fraction in each sample was estimated by measuring the sample absorbance (*A*_260_/*A*_280_) with a spectrophotometer (Lambda 6 UV-VIS; Perkin-Elmer, Buckinghamshire, UK). RNA reverse transcription to cDNA was performed on 1 μg of RNA using a QuantiTect Reverse Transcription kit (Qiagen). The samples were then stored at –80 °C until real-time PCR analysis.

The three genes whose expression was chosen to be studied as influenced by water stress and recovery were: *OePIP1.1* (Secchi *et al.*, 2007*b*; GenBank accession no. DQ202708) and *OePIP2.1* (Secchi *et al.*, 2007*b*; GenBank accession no. DQ202709), encoding functional water-channel proteins in olive; and the gene encoding an olive CA enzyme located in the chloroplast stroma (GenBank accession no. FN814304). We constructed a multiple alignment of the whole *Arabidopsis* family reported by Fabre *et al.* (2007), including the olive CA. The isoform of CA of olive was confirmed to be aligned with *Arabidopsis* stroma isoforms βCA (see Supplementary Fig. S4 at *JXB* online) described by Fabre *et al.* (2007) to be targeted to the chloroplasts. The CA full DNA coding sequence of *Arabidopsis thaliana* was retrieved from phytozome (http://www.phytozome.net) for phylogenetic analysis with the identified *O. europaea* mRNA (CA, GenBank accession no. FN814304). Alignment and phylogenetic analysis were performed with the seaview toolkit (Gouy *et al.*, 2010). Multiple alignment of mRNA sequences was performed using the MUSCLE algorithm (Edgar, 2004). As phylogenetic inference could be biased by partial sequences in the alignment, as with the *O. europaea* partial mRNA in our dataset, prior to phylogenetic analysis we extracted the evolutionary and conserved aligned blocks from the alignment using the Gblock algorithm (Talavera and Castresana, 2007). A phylogenetic tree was created by the maximum-likelihood approach using the PhyML algorithm (Guindon *et al.*, 2010), with the K80 (Kimura, 1980) nucleotide substitution model, 100 random tree starts, and an optimized ratio between nucleotide transition and transversion (*T*_s_/*T*_v_ ratio). Branch support was estimated by an approximate likelihood ratio test based on the Shimodaira–Hasegawa-like procedure (Shimodaira and Hasegawa, 1999; Guindon *et al.*, 2010).

Despite the fact that both *OePIPs* are more expressed in roots and twigs, we studied them because they are also expressed in leaves and are actually the only *PIPs* that have been studied and characterized in olive (Secchi *et al.*, 2007*b*). We included the β-actin gene (GenBank accession no. AF545569) as a housekeeping control. Specific primers were designed by Applied Biosystems (Foster City, CA, USA). The gene expression assays consisted of a 20× mix of unlabelled PCR primers and TaqMan MGB probes (labelled with carboxyfluorescein dye).

Real-time PCR (7300 Real-Time PCR System; Applied Biosystems) was performed on the samples, and relative gene expression was determined using the relative standard curve method (Applied Biosystems). As a calibrator, a sample from the experiment (C plant) was chosen, the calibration curves being performed with a 1, 10, 50, and 100ng dilution of the cDNA of the same sample, for each of the genes chosen to be studied plus the housekeeping gene. Fast PCR cycles were performed running 96-well Fast reaction plates with 20 µl of the reaction mix in every well and using TaqMan Universal PCR Master Mix (2×; Applied Biosystems). The PCR thermal cycling conditions used were the default ones, as indicated by the manufacturer’s instructions.

### Statistical analysis

Statistical analysis was carried out using Statistica for Windows v.6.0 software package (StatSoft). Significant differences between means were assessed using one-way analysis of variance applying Tukey’s test (*P*<0.05). Previously, data were tested for normality and homoscedasticity.

A d-separation method of path analysis was used to test several conceptual models that could explain the covariance among the measured physiological variables, especially *g*_m_, and the genetic expression of AQPs and CA. The proposed models were based on previous knowledge and the hypothesis of interactions between variables. Causal relationships between variables were combined to form directed graphs (the path models). These directed graphs implied a series of independence relationships between pairs of variables. Pearson correlation coefficients between pairs of variables were determined. The relationships among variables were then translated into a structure of variances and covariances that could be tested against the observed data. Path analysis was performed using the Causal Toolbox (Shipley, 2000) packages.

## Results

### Stress period

**Fig. 1.**
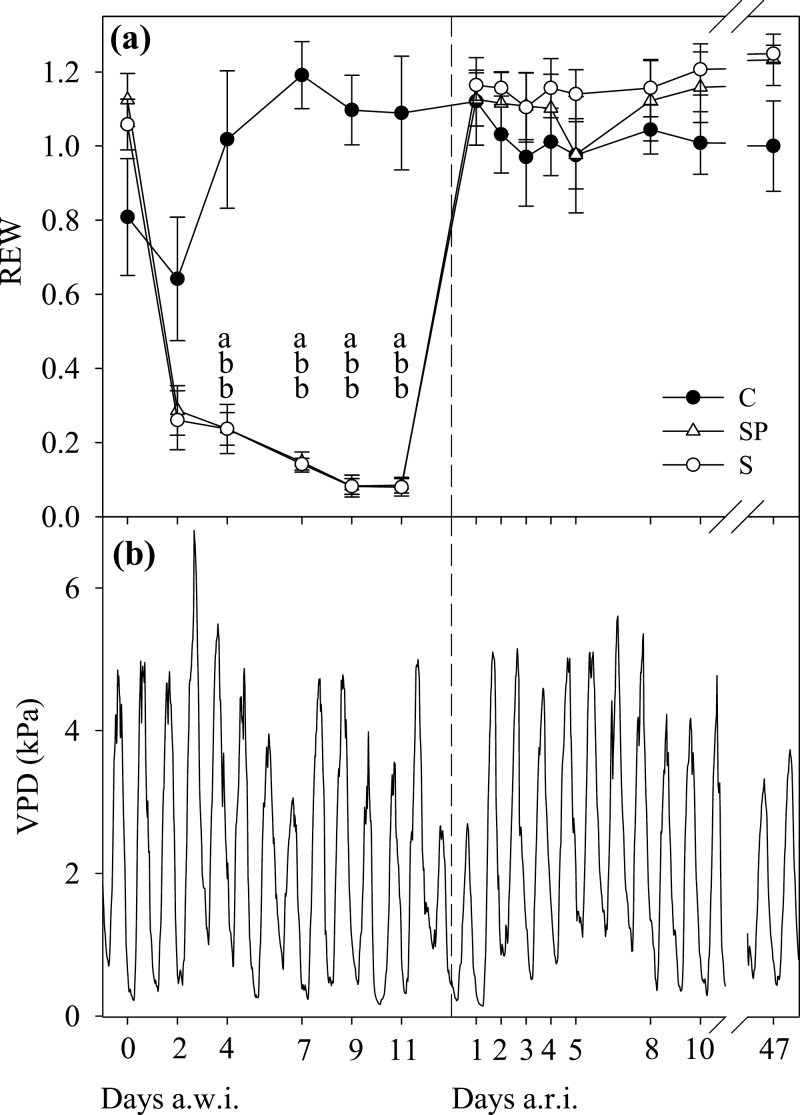
(a) Soil REW (see equation in Materials and methods) throughout the experiment in pots of the three treatments: C, control plants; S, stress plants; SP, stress-pruned plants. Values on each date are the means (±SE) between two different depths (0.05 and 0.20 m). Different letters indicate significant differences among treatments within each date (analysis of variance, Tukey: *P*<0.05). (b) VPD of air during the experiment in July and August of 2009. S and SP plants were last irrigated on day 0 a.w.i., with daily irrigation being applied again 13 d later (discontinuous line). a.w.i., after withholdong irrigation, a.r.i., after resuming irrigation.

**Fig. 2.**
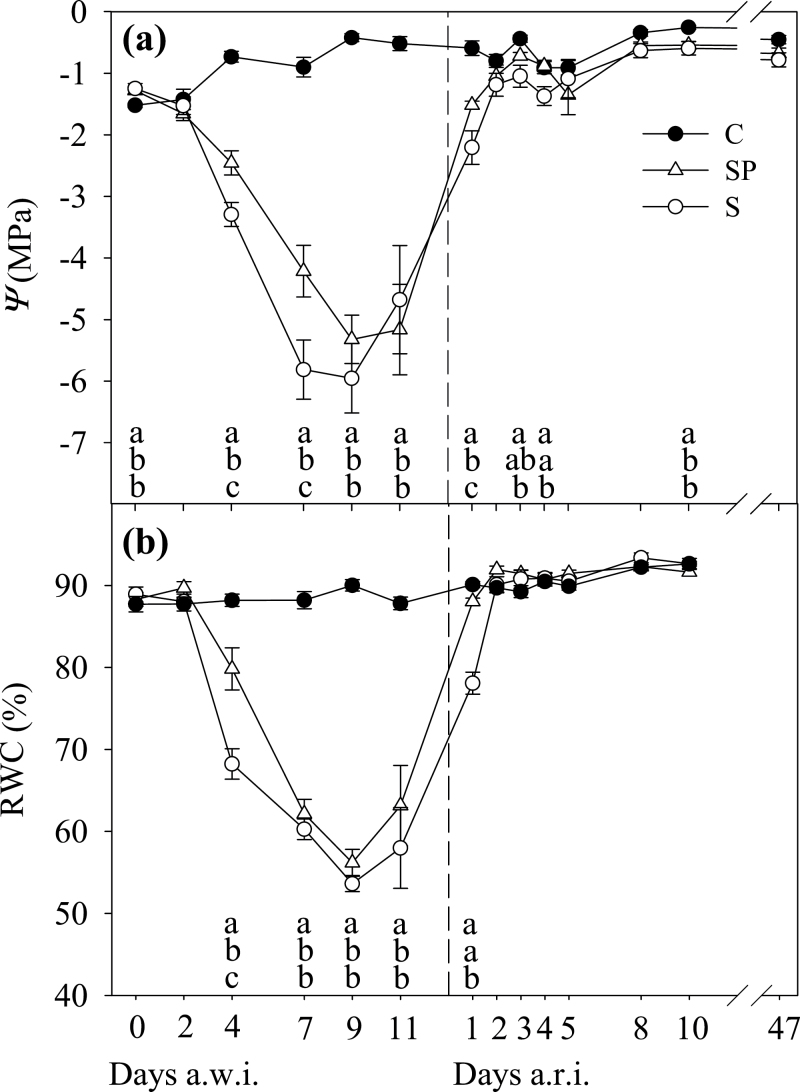
Leaf water potential (*Ψ*) (a) and RWC (b), both at 11.00 GMT, in olive plants throughout the experiment. Values are means (±SE) of six replicates per treatment and date. Treatments, letters, and the discontinuous line as described in Fig. 1.

**Fig. 3.**
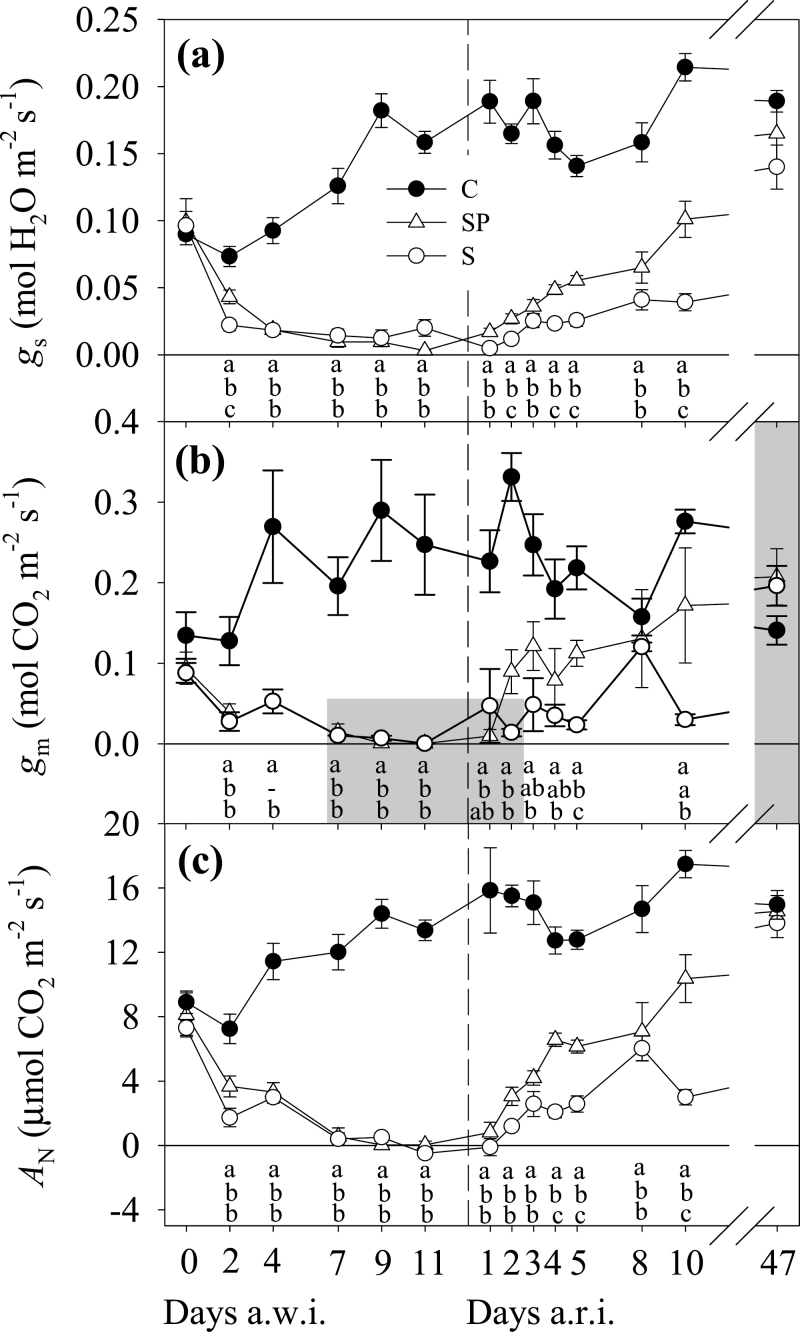
Time course during the drought period and the subsequent resumed irrigation of stomatal conductance to H_2_O (*g*_s_) (a), mesophyll conductance to CO_2_ (*g*_m_) (b), and net rate of CO_2_ assimilation (*A*_N_) (c), all measured at 11.00 GMT. Values are means (±SE) of four to six replicates per treatment and date. Unshaded areas indicate *g*_m_ data with a d*C*_c_/d*A*_N_ of between 10 and 50, which is reliable according to Harley *et al.* (1992). Treatments, letters, and the discontinuous line are as described in Fig. 1.

**Fig. 4.**
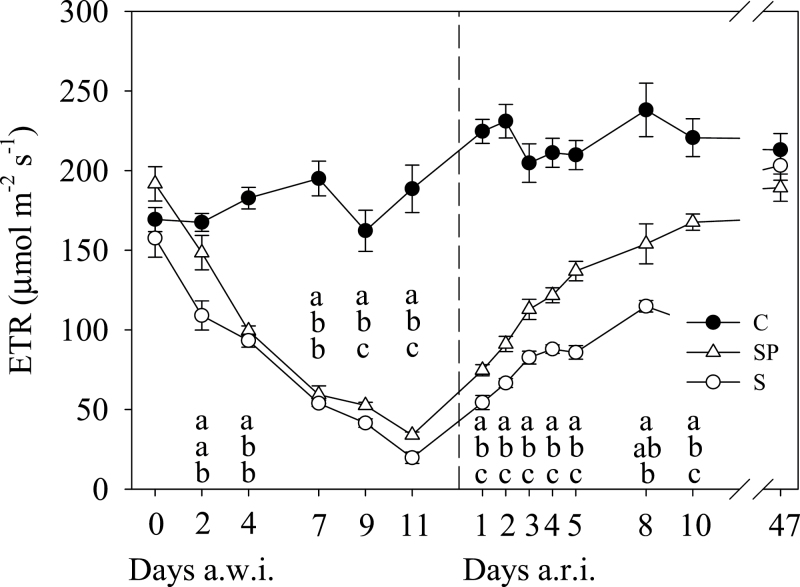
ETR at 11.00 GMT throughout the experiment. Each value is the mean (±SE) of six replicates per treatment and date. Treatments, letters and discontinuous line as described in Fig. 1.

REW dropped rapidly after water withholding in the S and SP treatments (Fig. 1a). Both stress treatments showed REW of <0.3 as soon as 2 d after withholding irrigation (a.w.i.). After that, a gentle decrease in REW down to 0.08 was recorded, suggesting very little water use by the plants. On average, the atmospheric demand for water was high, with maximum VPD values usually >4 kPa, and even reaching 6.8 kPa (Fig. 1b). During the initial drop in REW, both S and SP plants maintained both *Ψ* and RWC values similar to the C plants (Fig. 2), although with slight larger values for SP. However, differences with C were observed on day 4 a.w.i. and afterwards, with higher values in SP (with a smaller leaf area) than in S. At the end of the stress period, both stress treatments reached similar minimum values of *Ψ* and RWC of approximately –5.5MPa and 55%, respectively. Leaf gas-exchange variables were more responsive to the decline in soil REW, and a reduction in all three variables, *g*_s_, *g*_m_, and *A*_N_, was observed before changes in leaf water status were noticeable (Fig. 3). In parallel, ETR showed a continuous decrease in both S and SP plants from the beginning (Fig. 4).

**Fig. 5.**
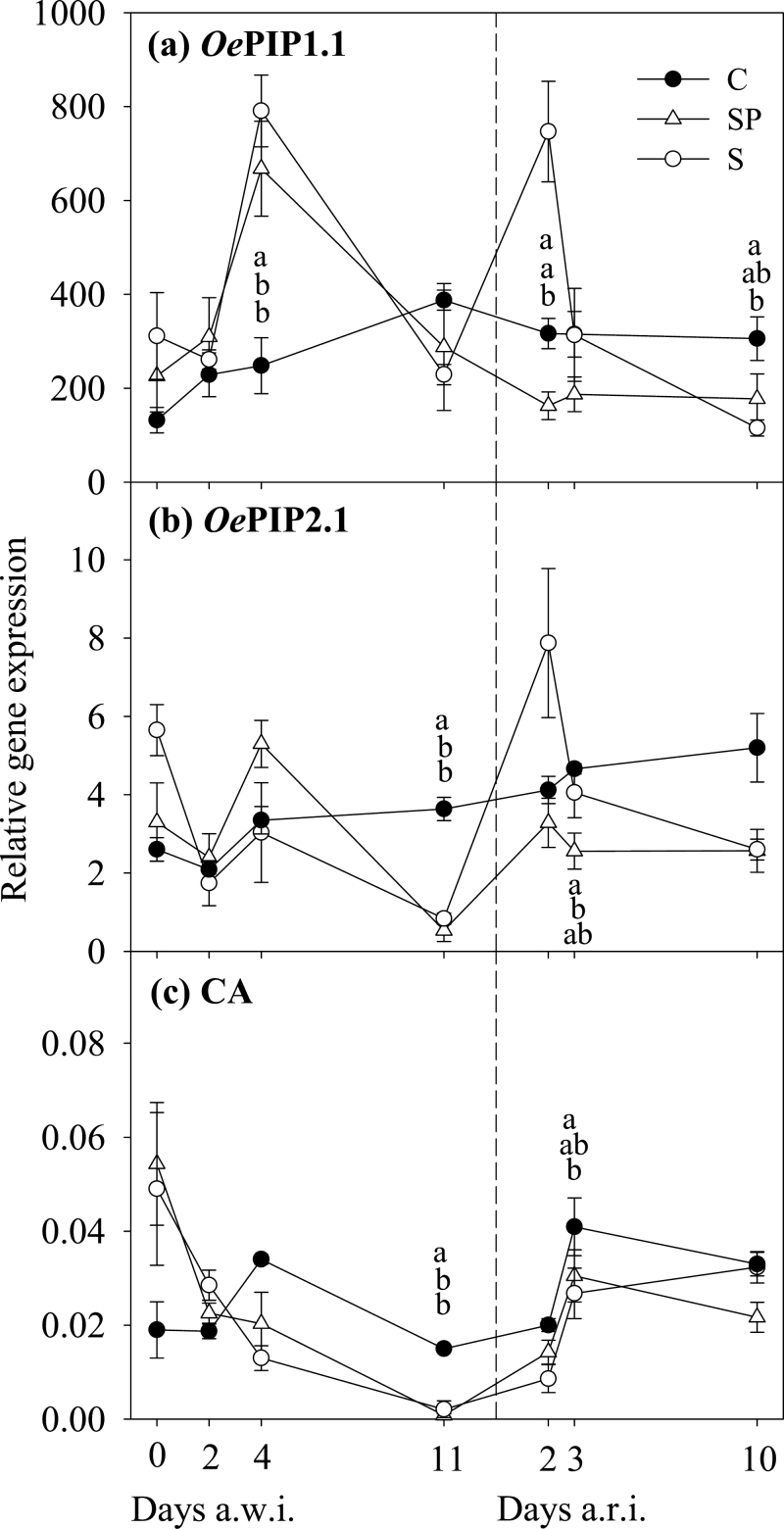
Time course of the relative gene expression of aquaporins OePIP1.1 (a) and OePIP2.1 (b), and CA (c) in leaves of *O. europaea* throughout the experiment. Values are means (±SE) of three replicates per treatment and date. Treatments, letters, and the discontinuous line are as described in Fig. 1.

*OePIP1.1* expression in S and SP plants peaked on day 4 a.w.i. and returned to C values at the end of the drought period (Fig. 5a). Expression of *OePIP2.1* in stressed plants was similar to that in C plants at the beginning of the drought period but diminished at the end (Fig. 5b). For CA, relative expression in stressed treatments was similar to that in C plants during the first days of drought but diminished at the end of this period (Fig. 5c). However, compared with the first day, relative expression in stressed treatments showed a continuous decrease from the beginning, similar to that described for ETR (Fig. 4).

### Recovery period

After resuming irrigation (a.r.i.), leaf water status recovered before gas-exchange variables did. At just 8h a.r.i., once REW was replenished (Fig. 1a), *Ψ* experienced a large recovery up to values around –2.0MPa, with higher values in SP than in S plants (Fig. 2a). On day 2 a.r.i., RWC recovered fully in S and SP plants, while *Ψ* did on days 3 and 5 a.r.i. in the case of SP and S plants, respectively. However, *g*_s_, *g*_m_, *A*_N_, and ETR had not reached C plant values in S and SP plants by day 10 a.r.i. In view of these results, another experimental day was programmed on day 47 a.r.i. (Figs 3 and 4), which finally confirmed the recovery in *g*_s_. In all variables, SP plants showed a larger capacity for recovery than S plants insofar as, on day 4 a.r.i., *g*_s_ in S and SP plants had recovered by 6 and 35%, *g*_m_ had recovered by 35 and 53%, and *A*_N_ had recovered by 20 and 53%, respectively. *OePIP1.1* expression did not change significantly after the recovery irrigation, except for a peak observed on day 2 a.r.i. in S plants, similar to that found during the first stage of the stress period. After this, all three treatments behaved similarly. In contrast, *OePIP2.1* expression presented a clear enhanced expression after irrigation, the response being stronger in S than in SP plants. CA expression recovered on day 3 a.r.i. (Fig. 5c).

### Photosynthesis limitations

**Fig. 6.**
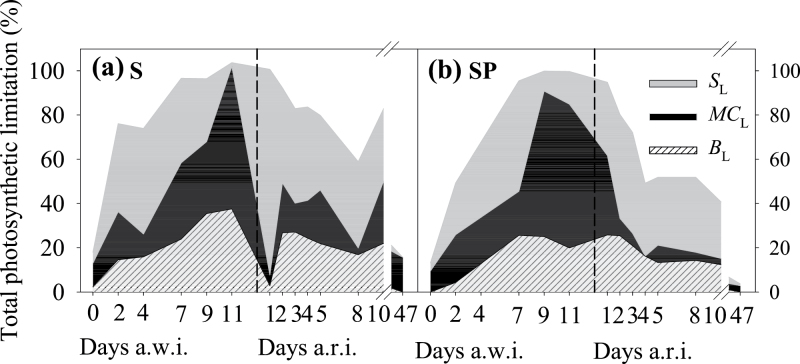
Quantitative limitation analysis of photosynthesis during the drought period and the subsequent resumed irrigation in S (a) and SP (b) plants. The shaded areas represent the percentage of stomatal (*S*_L_), mesophyll conductance (*M*C_L_), and biochemical (*B*_L_) limitation based on C plants on each date. Values are means of four to six replicates per treatment and date. S and SP plants were last irrigated on day 0 a.w.i., with daily irrigation being applied again 13 d later (discontinuous line).

During the drought period, the diffusional limitations (*D*_L_=*S*_L_+*MC*_L_) prevailed over *B*_L_ (Fig. 6). Two main stages in the time course of photosynthesis limitations could be differentiated in both S and SP treatments. In the initial one, during the first 2–4 d the drop in *g*_s_ was accompanied by a predominant role of *S*_L_ accounting for up to 50% of the *T*_L_ in S and 35% in SP plants. In the second one, during the next 9 d without irrigation where *B*_L_, and especially *MC*_L_, took the dominant role, *S*_L_ lost importance. During this stage of severe water stress, *B*_L_ was around 20% of *T*_L_. At the end of the drought period, all limitations in S plants were imposed by *MC*_L_ and *B*_L_, accounting for 35 and 65%, respectively. Similar trends were observed in SP plants, with a minimal role of *S*_L_ (17%).

During the recovery period, *D*_L_ also prevailed over *B*_L_. The dynamics of the limitations during this period were different between the stressed treatments. In S plants, on 1 d a.r.i. nearly 100% of *T*_L_ was *S*_L_, and it then accounted for about 40% until the end of the experiment. The main difference observed between S and SP plants was that, in the latter, S_L_ was not so exclusive on the first day of recovery, with *MC*_L_ and *B*_L_ playing a significant role. On the following days, T_L_ was between 20 and 30% lower in SP than in S plants, with a significantly lower *MC*_L_. All limitations had disappeared by d 47 a.r.i.

### Relationships between variables and causal models

**Fig. 7.**
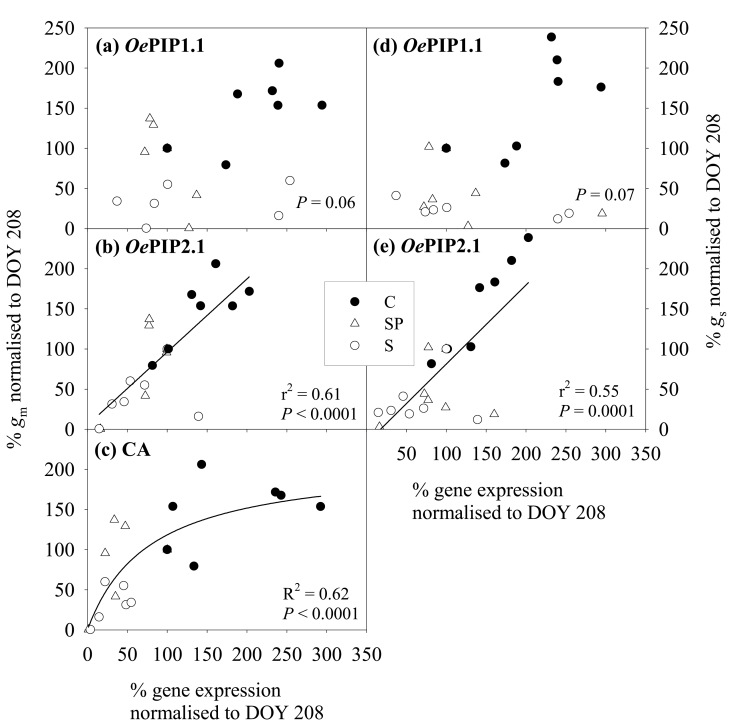
Relationships between *g*_m_ and gene expression (a, *OePIP1*.1; b, *OePIP2*.1; c, CA) and between *g*_s_ and gene expression (d, *OePIP1.1*; e, *OePIP2.1*), all normalized to the first day of experiment (26 July). Values are means on each date and treatment (*g*_m_ and *g*_s_: *n*=6; gene expression: *n*=3). Treatments are described in Fig. 1.

Normalizing the gene expression, *g*_s_, and *g*_m_ by the first date (26 July) allowed us to eliminate the factor of different absolute values and concentrate on the evolution along the experiment. There were linear positive relationships of *g*_m_ (*P*=0.0001) and *g*_s_ (*P=*0.0001) with the expression of *OePIP2.1* (Fig. 7b, e) and a hyperbolic one between *g*_m_ and the expression of CA (*P*<0.0001) (Fig. 7c). Although *OePIP1.1* did not show a significant relationship with *g*_m_ and *g*_s_, this was largely due to the inclusion of the peak points measured just after the imposition of water stress or just after irrigation was resumed. If we discarded these values (on the bottom right corner of Fig. 7a, b), we obtained a significant correlation between variables in both cases (*P=*0.003, *r* ^2^=0.43, and *P*<0.0001, *r* ^2^=0.64, for *g*_m_ and *g*_s_, respectively).

**Fig. 8.**
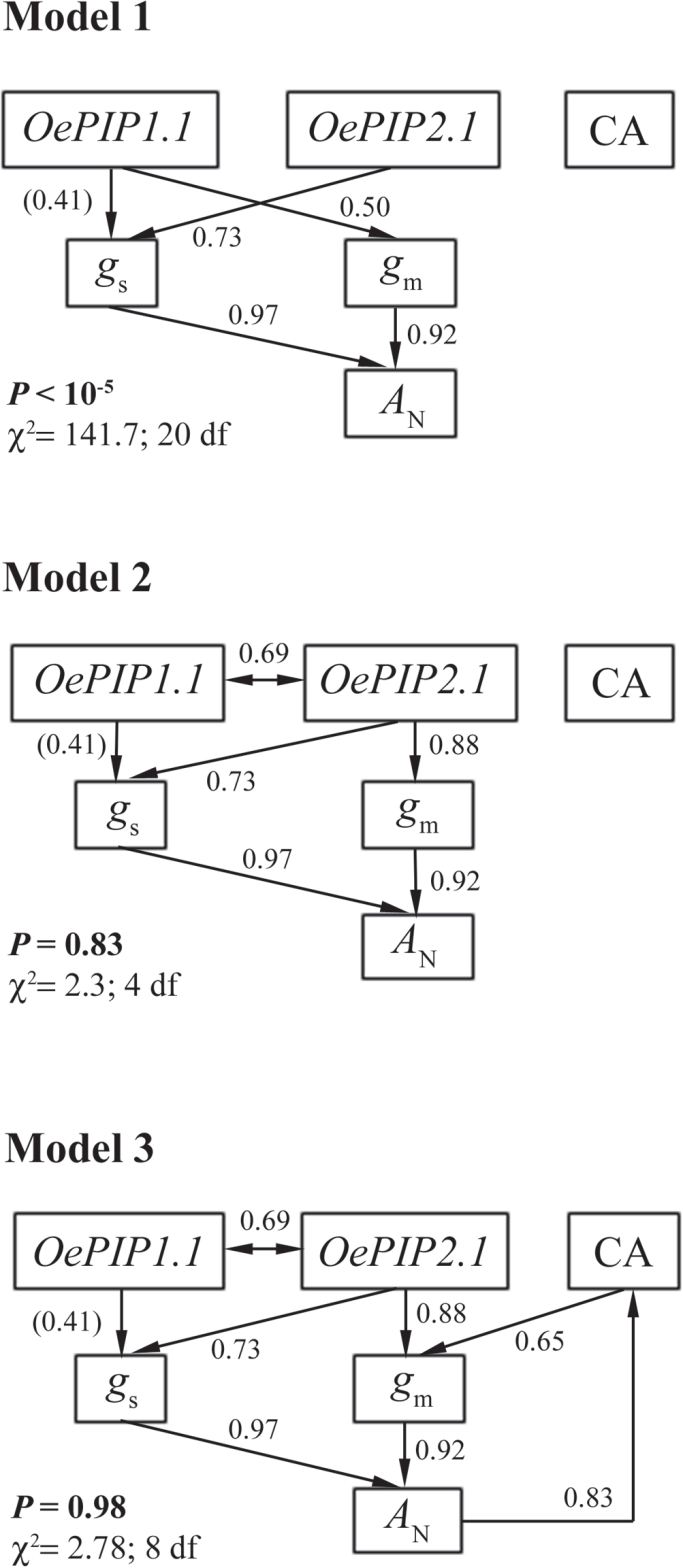
Examples of the alternative causal models tested using the d-separation method to describe causal relationships among photosynthetic (*g*_s_, *g*_m_, and *A*_N_) and molecular variables (genetic expression of *OePIP1.1*, *OePIP2.1*, and CA). Arrows and numbers close to them indicate direct relationships and correlation coefficients, respectively, between variables (in brackets are the non-significant correlation coefficients).

Several alternative models were performed and studied with a path analysis to reveal the causal relationships that linked gene expression with *g*_m_, *g*_s_, and *A*_N_. Of all the possible combinations among variables, only those that were physiologically sound were tested. We stated *a priori* the relationships among variables with a strong mechanistic or well-established and accepted empirical basis only (Shipley, 2000). The main underlying hypotheses were: (i) *A*_N_ is determined mainly by *S*_L_+*MC*_L_ (Grassi and Magnani, 2005; Diaz-Espejo *et al.*, 2007); (ii) AQPs can affect H_2_O fluxes (*g*_s_) (Secchi *et al.*, 2007*b*; Laur and Hacke, 2013); (iii) AQPs can affect CO_2_ fluxes (*g*_m_) (Heckwolf *et al.*, 2011; Kawase *et al.*, 2013); (iv) PIP1.1 and PIP2.1 interact to modify the traffic to the membranes and to build a tetramer that confers the basis of the CO_2_ transport function (Zelazny *et al.*, 2007; Otto *et al.*, 2010); and (v) CA can affect *g*_m_ in sclerophyll plants (Gillon and Yakir, 2000). Fig. 8 shows three of the most representative models tested. These three were consistent with hypotheses 1 and 2. Model 1 included the role of *OePIP1.1* in *g*_m_, reported for other PIP1s in other species, but, even though CA was included affecting *g*_m_ directly, this model was rejected on the grounds that *P* was <0.05 (i.e. it had little prediction capacity). Nevertheless, when in model 2, we changed the role of *OePIP1.1* in *g*_m_ for that of *OePIP2.1* and included the interaction between *OePIP1.1* and *OePIP2.1*, simulating the tetramer structure, we obtained a satisfactory explanation for a high proportion of the variance found (*P*=0.83). However, the best fit to the data was reached with model 3, which completed model 2 by incorporating the role of CA, as a function of *A*_N_, in *g*_m_ (*P*=0.98). The inclusion of *Ψ* (as a surrogate of plant water stress) affecting *OePIP1.1* and *OePIP2.1* reduced the proportion of the variance found (*χ* ^2^=12.92; *P*=0.93), although the resulting model continued to be useful.

## Discussion

### Physiological response to water stress and recovery

Photosynthetic variables were the first to respond to irrigation withholding, being lower in stressed treatments than in C plants on d 2 a.w.i. (Figs 3 and 4). Leaf water status responded 2 d later (Fig. 2). The initial reduction in *g*_s_, larger in S than in SP plants, was enough to keep *Ψ* at constant values, demonstrating the fine control of plant water status in olive by stomata at the initial stages of water stress (Figs 2a, and 3a) (Fernández *et al.*, 1997; Torres-Ruiz *et al.*, 2013*a*). Stomatal closure has been interpreted as a plant mechanism to maintain xylem *Ψ* well above a critical value avoiding catastrophic hydraulic failure (Tyree & Zimmermann, 2002). On d 4 a.w.i., *Ψ* was around –3.0MPa, corresponding to a xylem *Ψ* around –5MPa, which is not enough to provoke important losses of hydraulic conductivity in olive (around 15–30% loss of hydraulic conductivity), as some works have reported (Ennajeh *et al.*, 2008; Diaz-Espejo *et al.*, 2012). However, at this stage, *g*_s_ had nearly reached its minimum value, and there was no further capacity to control *Ψ*, which later dropped to very low values of –6MPa in the case of S treatment. The progress of stress in the experiment was quicker than we expected initially. Although we used large pots (50 l), the leaf area and the high atmospheric demand in our experimental site meant that plants consumed the available water in a few days. The reduction in leaf area by pruning was not enough to make large differences between both stressed treatments, but some significant differences were still found between them as shown in Figs 3 and 4, 2 d a.w.i. and especially a.r.i. The time of the day at which *Ψ* and leaf gas exchange measurements were made were chosen carefully to try to determine the most significant moments: minimum *Ψ* and maximum *g*_s_. The lack of difference in *Ψ* between treatments on many days, as a consequence of its isohydric behaviour, did not affect the difficulty of unravelling causal relationships, although certainly it would be desirable to repeat the experiment under a more gradual drought-stress imposition in the future.

After rewatering, leaf water status recovered earlier than photosynthetic variables from drought, matching the C plant values on d 5 a.r.i., while *A*_N_ and ETR did so on d 10 a.r.i. (Figs 2–4). Olive has a high capacity for recovery from water stress after rewatering, as previous studies have shown (Moriana *et al.*, 2007; Torres-Ruiz *et al.*, 2013*b*). A similar pattern has been reported in tobacco (Galle *et al.*, 2009). The value of *g*_s_ showed the slowest recovery of all the variables studied. The reasons for this commonly slow recovery of *g*_s_ after restoration of leaf water status is not yet clear, although it has been related to both hydraulic limitations (Brodribb and Cochard, 2009) and chemical limitations (Lovisolo *et al.*, 2008). The above-mentioned low values of *Ψ* reached at the end of the drought period (approx. –6MPa) suggested that a large loss of stem hydraulic conductivity could be a hydraulic factor conditioning the slow and partial recovery of *g*_s_ when water was applied during the recovery stage. However, even at these values of *Ψ*, olive is not expected to lose much of its hydraulic conductivity: between 35 and 40% according to Ennajeh *et al.* (2008), explaining the ability of this species to tolerate severe drought (Torres-Ruiz *et al.*, 2013*b*). Other components of the hydraulic system could have not recovered after resuming irrigation and would have been limiting, like leaves or roots. However, this does not look to be the case, as reported recently by Torres-Ruiz *et al.* (2013*b*). Concerning chemical limitations, several authors have pointed out the accumulation of abscisic acid (ABA) during stress acclimation as a circumstance that might prevent *g*_s_ from fully recovering once water is available again and plant water status has been restored (Davies and Zhang, 1991; Lovisolo *et al.*, 2008). Lovisolo *et al.* (2008) explained this role played by ABA in the recovery stage as a way of controlling transpiration rate during the embolism-repairing time. However, the conclusions of that work, carried out in grapevine, are not necessarily applicable to olive, especially as the former has been reported to have a great refilling capacity of embolized vessels (Brodersen *et al.*, 2010). Moreover, even in grapevine, contradictory results have been obtained, with no recovery of *g*_s_ once ABA accumulation was fully reversed after a few days (Pou *et al.*, 2008). Finally, several authors have reported that recovery from water stress depends on the level and velocity of the stress imposition (Niinemets *et al.*, 2009; Galle *et al.*, 2011). In this sense, our experiment showed a faster recovery of all physiological variables in SP than in S plants.

Our results confirm the hypothesis that *D*_L_ prevails under most water-stress situations in numerous species (Flexas *et al.*, 2002; Keenan *et al.*, 2010) and that *B*_L_ usually appears only under severe water stress (Flexas *et al.*, 2006*a*; Galmés *et al.*, 2007*a*) (Fig. 6). Although during the first 2–4 d of drought *S*_L_ was the only prevailing limitation, as drought intensified to a severe level both *MC*_L_ and *B*_L_ increased. *MC*_L_ in particular accounted for up to 60% of the *T*_L_, reflecting the important and determinant role of *g*_m_ in the photosynthesis rates. This agrees with previous reports in similar experiments (Galmés *et al.*, 2007*a*; Galle *et al.*, 2011) where, under the most severe conditions of water stress *MC*_L_ proved to be the maximum limitation. Once water was available again after irrigation, the most responsive variable determining the recovery of *A*_N_ was *g*_m_. *MC*_L_ was reduced to a residual 15–20% in the S treatment, while it was nearly eliminated in the SP treatment (Fig. 6). The faster recovery of photosynthetic variables in SP compared to S plants translated into lower values of *T*_L_ in SP than in S plants. A good correlation between *g*_s_ and *g*_m_ was found for most of the studied period with a *g*_m_:*g*_s_ ratio (both on CO_2_ basis) of 2.1. Considerable evidence of the close relationship between *g*_s_ and *g*_m_ has been reported (Flexas *et al.*, 2008; Warren, 2008*b*), but this relationship has been regarded as the result of a tight covariance of these two resistances in the CO_2_ pathway to the leaf rather than as a cause-effect relationship. In fact, several works have indicated that the relationship between *g*_s_ and *g*_m_ can be modified according to changing environmental conditions (Flexas *et al.*, 2009; Galle *et al.*, 2009 Perez-Martin *et al.*, 2009; Flexas *et al.*, 2013*a*).

### Gene expression of AQPs and CA during drought and recovery

The expression patterns during stress treatments were different between the two AQPs studied and were more irregular than for CA (Fig. 5). This impression was shown by the peak responses measured at the beginning of both stress and recovery periods in *OePIP1.1*. The peaks at the onset of drought have been reported in similar studies (Yamada *et al.*, 1997; Galmés *et al.*, 2007*b*; Pou *et al.*, 2013), where the upregulation of AQP expression, including *PIP1* and *PIP2*, was interpreted as a mechanism to promote water movement inside leaves via symplast by increasing membrane permeability to water when this is less available for the plant. The peak of expression in *OePIP1.1* at the start of the re-irrigation period could explain the fast recovery of leaf water status by the possible role of AQPs in the xylem refilling of parenchyma cells (Kaldenhoff *et al.*, 2008). This peak would occur in a moment of high water availability in soil, which would reduce risks of massive losses of water from leaves (Secchi *et al.*, 2007*a*). On the other hand, the downregulation in *OePIP2.1* expression at the end of drought period may encourage cellular water conservation during periods of water stress by reducing membrane water permeability and limiting loss of cellular water (Secchi *et al.*, 2007*a*). Probably, in order to maintain a suitable water status under abiotic stress, both increased water transport via AQPs in some tissues and reduced in others are required (Secchi *et al.*, 2007*b*) because one of the main role of AQPs is to maintain homeostasis and water balance under water-stress conditions (Tyerman *et al.*, 2002).

In addition to their role in water transport, there is published evidence of the putative role of AQPs in CO_2_ transport and *g*_m_ (Uehlein *et al.*, 2003; Kawase *et al.*, 2013). Accordingly, we found a linear relationship between *OePIP2.1* expression and *g*_m_, but no relationship between *OePIP1.1* expression and *g*_m_ (Fig. 7). Although it is found that most members of PIP2 act as water channels (Tsuchihira *et al.*, 2010) while PIP1 members are involved in a CO_2_ transport function (Flexas *et al.*, 2006*b*; Heckwolf *et al.*, 2011), Hanba *et al.* (2004) found differences in *g*_m_ induced by a PIP2. On the other hand, it has been reported that PIP1 and PIP2 AQPs form heterotetramers, which modify their function as H_2_O or CO_2_ membrane transport facilitators depending on their composition (Otto *et al.*, 2010); hence, both PIP1 and PIP2 may be inextricably linked with each other and with *g*_m_. On the other hand, *OePIP2.1* showed a similar degree of correlation with *g*_s_, as already observed for *PIP2.1* of grapevines (Pou *et al.*, 2013). In summary, while it is unclear from the data whether the expression of specific AQPs is related to regulation of both *g*_s_ and *g*_m_, or whether they just operate by affecting one of the two conductances and a tight co-regulation between *g*_s_ and *g*_m_ provokes an apparent correlation of the other with *PIP* expression, the results strongly suggest that AQP expression is involved in setting diffusional limitations to photosynthesis in olives under water stress and recovery. This same link provokes an additional tight correlation between the two apparently less related parameters of *g*_m_ and the leaf hydraulic conductance (Flexas *et al.*, 2013*b*).

The CA expression patterns in stressed treatments throughout the experiment were smoother than AQP patterns and were similar in S and SP plants (Fig. 5c), closely tracking those observed for ETR (Fig. 4). The role of this stroma CA in *g*_m_ regulation was supported by the relationship found between CA expression and *g*_m_ (Fig. 7c). The hyperbolic shape of this relationship agrees with the consideration of CA as not being limiting to photosynthesis due to the large amount of CA present in leaves, although this can be species dependent (Makino *et al.*, 1992). Although the role of CA in regulating *g*_m_ is more controversial than the evidence found for AQPs (Flexas *et al.*, 2012), some authors have reported that this role can be species dependent, gaining importance when *g*_m_ is low, as happens in sclerophyll species (Gillon and Yakir, 2000). Therefore, this would also be the case for olive, a sclerophyllous species with a thick cell wall (Bacelar *et al.*, 2004; Marchi *et al.*, 2008). Following the results of Gillon and Yakir (2000), olive would have to counterbalance the low conductance at the cell-wall sites with a larger conductance at the chloroplast sites, which undoubtedly would make *g*_m_ more dependent on CA.

To analyse the causal nature and the structure of the relationships found here (Fig. 7b, c), we considered several causal models with physiological sense that fitted the data. The most robust structure with the highest significant levels in many of the combinations tested was that obtained in model 3 (Fig. 8). This model showed that there could be a direct role of *OePIP2.1* and CA expression in *g*_m_ regulation, as well as an indirect role of OePIP1.1. The interaction between both AQPs is justified in accordance with the conclusions by Otto *et al.* (2010), who suggested that PIP1 and PIP2 interact to form a heterotetramer. Additionally, the model considers that both PIPs are directly involved in the regulation of *g*_s_, that both *S*_L_ and *MC*_L_ determine *A*_N_, and that CA is in turn regulated by *A*_N_. *OePIP1.1* and *OePIP2.1* in olive have been described to regulate membrane water permeability in leaves (Secchi *et al.*, 2007*b*), a fact that explains their effect on *g*_s_. CA was tested in an independent model (not shown) to regulate *g*_m_ directly without the role of AQPs, this model being able to explain only a small amount of the data variance (*χ* ^2^=22.53; degrees of freedom=16; *P*=0.126). This supports the hypothesis that the role played by CA in the regulation of *g*_m_ is not central. In any case, in addition to the implications in sclerophyllus leaves mentioned above (Gillon and Yakir, 2000), the inclusion of CA as a function of *A*_N_ is justified because the regulation of CA gene expression is related to the leaf photosynthetic activity by the CO_2_ inside leaves (Hoang and Chapman, 2002; Fabre *et al.*, 2007). CA has been reported to be closely related to Ribulose-1,5-bisphosphate carboxylase/oxygenase (Rubisco) activity, and therefore to *A*_N_ (Makino *et al.*, 1992; Price *et al.*, 1994). Furthermore, the time course of CA expression and *A*_N_ were quite similar throughout the experiment (Figs 5c and 3c) and recent studies have demonstrated the role of CA as an upstream regulator of CO_2_-controlled stomatal movements in guard cells (Hu *et al.*, 2010). While the validation of a causal model does not imply that the hypothesis is true, it does imply that the hypothesis is plausible given the empirical data (Shipley, 2000). *C*_i_ has long been proposed as a potential signal used by the plant to maintain the equilibrium among *g*_s_, *g*_m_, and *A*_N_ (Ethier *et al.*, 2006; Flexas *et al.*, 2009). Under water stress, downregulation of *g*_s_, *g*_m_, *V*_cmax_, and ETR could occur to keep *C*_i_ under relatively constant values during the acclimation process. If this hypothesis is correct, CA would be in an optimal position for regulation of g_m_, as it uses as *C*_i_ as a substrate and influences its diffusion inside the cell (Terashima *et al.*, 2011). Although gene expression may not necessarily reflect protein function because of the post-transcriptional regulation (Maurel, 2007; Heinen *et al.*, 2009; Laur and Hacke, 2013), the good relations of *g*_m_ with expression of *OePIP2.1* and CA and the good fitting of the causal model proposed allow us to go beyond already published literature and to infer a direct role of AQPs and CA in *g*_m_ regulation, although certainly the mechanistic basis for such a role remains to be elucidated.

## Conclusions

The main objective of this study was to provide a step forward in the putative role played by AQPs and CA in the regulation of *g*_m_ under water stress. Despite the use of the path analysis to infer some potential causal effects, a novel aspect of this work is the execution of the experiment outdoors under natural conditions. Photosynthetic variables in olive trees responded faster than water status under drought but recovered much later. Despite the severe stress, *D*_L_ prevailed over *B*_L_ throughout the experiment. The role of *MC*_L_ varied throughout the experiment, prevailing over the rest of the limitation components as stress intensified and being present even during the recovery phase. This study supports the hypothesis that the regulation of *g*_m_ is regulated mainly by AQPs and that both *OePIP1.1* and *OePIP2.1* are likely to interact to exert a significant effect on *g*_m_. The data shown in this study reveals novel evidence on the putative role of CA in the regulation of mesophyll conductance to CO_2_, which, although small if compared with that of AQPs, is justified by the sclerophyllous nature of olive leaves. Due to the tight correlation between AQPs and both *g*_s_ and *g*_m_, evidence of co-regulation between the two conductances and the fact that H_2_O and CO_2_ fluxes in leaves share a part of their pathways, facilitated by AQPs, it is very difficult to relate genes with functions in an unequivocal manner. As far as we know, there are few than expected studies showing this sort of correlation and, of course, no reverse genetics approach is possible to date with woody species like olive trees. Hence we believe that our approach, although certainly limited, is useful for a better understanding of plant physiological response to water stress, and is perhaps the only one currently available for olives. New biochemical probes and techniques are therefore required in order to quantify the activity of AQPs and their genetic expression changes, as well as to follow their spatial distribution in cells.

## Supplementary data

Supplementary data are available at *JXB* online.

Supplementary Fig. S1. Picture showing the aspect of the experimental set-up: 5-year-old trees in 50 l pots.

Supplementary Fig. S2. Relationship of the quantum yield of photosystem II (ΦPSII) and ΦCO^2^ [(*A*_N_+*R*_d_)/PPFD].

Supplementary Fig. S3. Relationship between *V*_cmax_ and ETR used in the quantitative analysis of photosynthesis limitations throughout the experiment.

Supplementary Fig. S4. Maximum-likelihood tree of *Olea europaea* carbonic anhydrases (CA) with *Arabidopsis thaliana* CA.

Supplementary method. Assessment of limitations to photosynthesis.

Supplementary Data

## Abbreviations:

ABA: abscisic acid
AQP: aquaporin
a.r.i.: after resuming irrigation
a.w.i.: after withholding irrigation
CA: carbonic anhydrase
*C*_i_: internal CO_2_ concentration
ETR: electron transport rate
GMT: Greenwich Mean Time
*g*_m_: mesophyll conductance to CO_2_
*g*_s_: stomatal conductance to CO_2_
PSII: photosystem II
REW: relative extractable water
RWC: relative water content of leaves
SE: standard error
VPD: vapour pressure deficit.
