# Supplementary material for: Regulation of photosynthesis and stomatal and mesophyll conductance under water stress and recovery in olive trees: correlation with gene expression of carbonic anhydrase and aquaporins
Source: J Exp Bot. 2014 May 5;65(12):3143–56. doi: 10.1093/jxb/eru160 (PMC4071832; doi:10.1093/jxb/eru160)
Supplement: Supplementary Data [file supp_65_12_3143__index.html]

Regulation of photosynthesis and stomatal and mesophyll conductance under water stress and recovery in olive trees: correlation with gene expression of carbonic anhydrase and aquaporins — Regulation of photosynthesis and stomatal and mesophyll conductance under water stress and recovery in olive trees: correlation with gene expression of carbonic anhydrase and aquaporins — Supplementary Data 

# Regulation of photosynthesis and stomatal and mesophyll conductance under water stress and recovery in olive trees: correlation with gene expression of carbonic anhydrase and aquaporins

## Supplementary Data

Data files

**Files in this Data Supplement:**

- Supplementary Data - Supplementary Data
