# Supplementary material for: Regulation of photosynthesis and stomatal and mesophyll conductance under water stress and recovery in olive trees: correlation with gene expression of carbonic anhydrase and aquaporins
Source: J Exp Bot. 2014 May 5;65(12):3143–56. doi: 10.1093/jxb/eru160 (PMC4071832; doi:10.1093/jxb/eru160)
Supplement: Supplementary Data [file supp_eru160_jexbot113605_file001.pdf]

Supplementary material

Regulation of photosynthesis, stomatal and mesophyll conductance under water stress and recovery in olive trees: correlation with gene expression of carbonic anhydrase and aquaporins

Alfonso Perez-Martin, Chiara Michelazzo, Jose M. Torres-Ruiz, Jaume Flexas, José E. Fernández, Luca Sebastiani, Antonio Diaz-Espejo

Assessment of limitations to photosynthesis

Grassi and Magnani (2005) suggested a new approach whose maximum innovation was the quantification of each of the main components affecting photosynthesis limitation; these are: stomatal, mesophyll conductance and biochemical. Each of these limitations were defined, following Jones (1985), combining a proper parameterization of the Farquhar *et al.* model of photosynthesis with estimates of stomatal and mesophyll conductance, and were described as

$$S_L = \frac{g_{tot} / g_{sCO_2} \cdot \partial A_N / \partial C_c}{g_{tot} + \partial A_N / \partial C_c}$$

$$MC_L = \frac{g_{tot} / g_m \cdot \partial A_N / \partial C_c}{g_{tot} + \partial A_N / \partial C_c}$$

$$B_L = \frac{g_{tot}}{g_{tot} + \partial A_N / \partial C_c}$$

where g_{tot} is total conductance to CO_2 between the leaf surface and carboxylation sites ($1/\ g_{tot} = 1/\ g_{sCO2} + 1/\ g_m$), g_{sCO2} is stomatal conductance to CO_2, g_m mesophyll conductance to CO_2, A_N net CO_2 assimilation rate, C_c chloroplastic concentration of CO_2, and S_L, MC_L and B_L are the stomatal, mesophyll conductance and biochemical limitations, respectively, with value between zero and one.

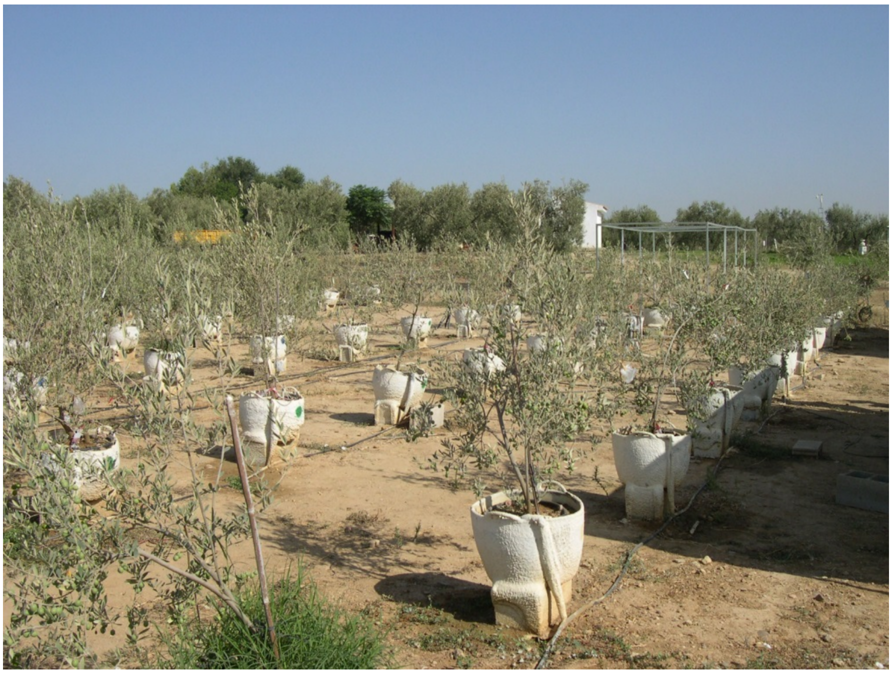

Figure S1. Picture showing the aspect of the experimental set-up, five-year-old trees in 50 L pots.

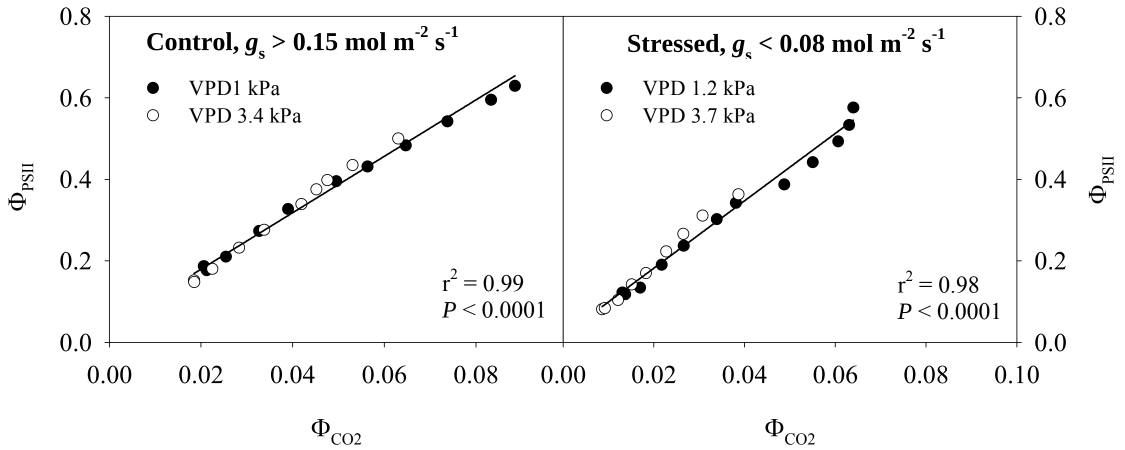

Figure S2. Relationship of the quantum yield of photosystem II (Φ_{PSII}) and Φ_{CO2} (A_N+R_d)/PPFD]. Measurements were carried out under low oxygen (<1%). These curves were performed on leaves under high air vapour pressure deficit (VPD) (open symbols), and low VPD (filled symbols), both under well-watered 'control' and re-watered conditions.

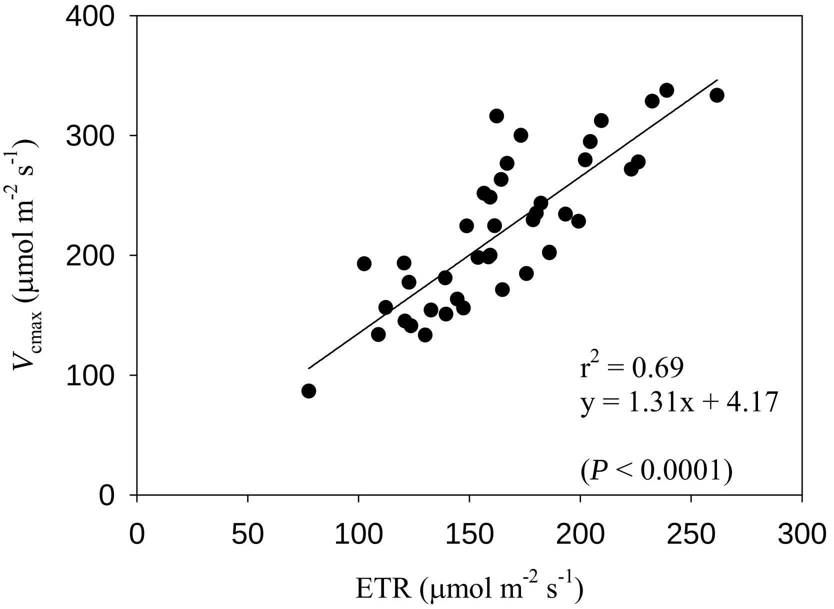

Figure S3. Relationship between $V_{cmáx}$ and ETR used in the quantitative analysis of photosynthesis limitations along the experiment.

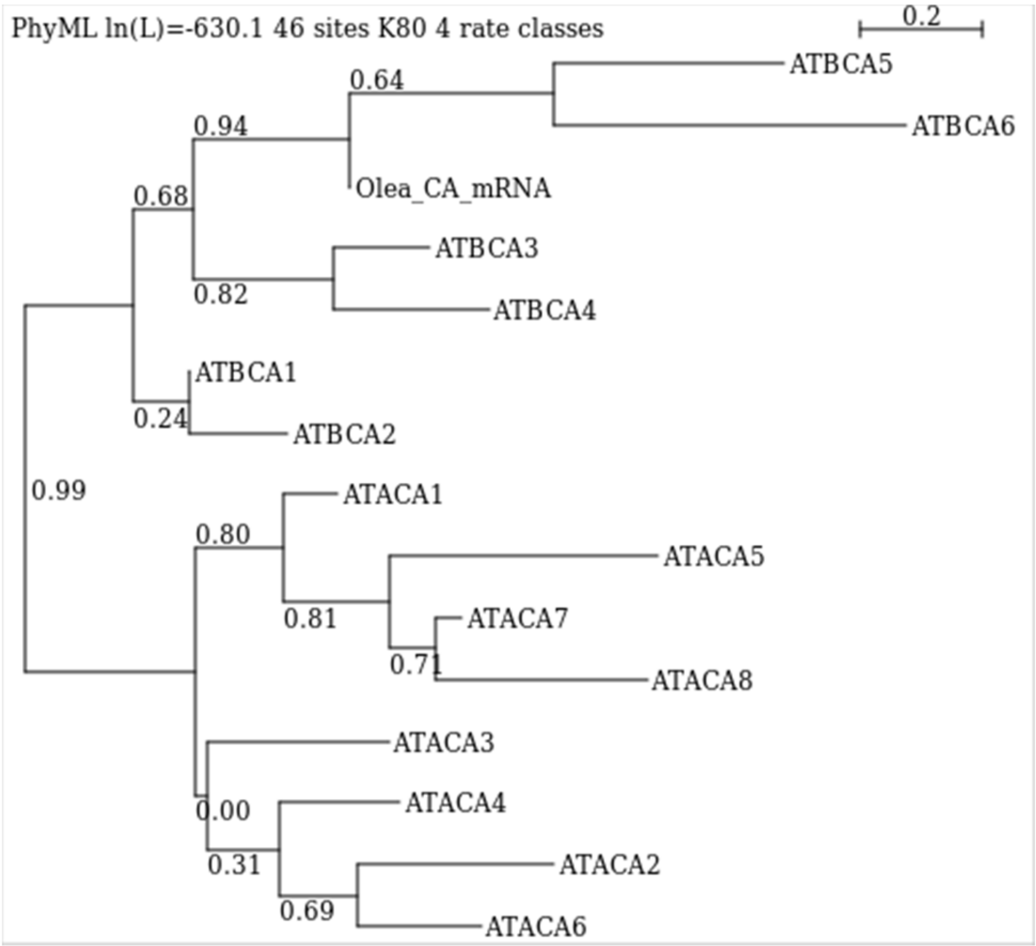

Figure S4. Maximum Likelihood tree of *Olea europaea* carbonic anhydrases (CA) with *Arabidopsis thaliana* CA. ATACA1-8 are α-type CA, ATABCA1-6 are β-type CA of *Arabidopsis*. Branch support values based on aLRT (SH-like) method are showed.
